# The Construction of Sham Dry Needles and Their Validity

**DOI:** 10.1155/2018/9567061

**Published:** 2018-06-14

**Authors:** Ulrike H. Mitchell, Paul Stoneman, Robert E. Larson, Garritt L. Page

**Affiliations:** ^1^Department of Exercise Sciences, Brigham Young University, Provo, UT 84601, USA; ^2^Department of Physical Therapy, Rocky Mountain University of Health Professions, Provo, UT 84606, USA; ^3^Department of Statistics, Brigham Young University, Provo, UT 84601, USA

## Abstract

Appropriate control interventions are necessary to show the treatment effect of dry needling. Different control procedures, such as dry needling of the contralateral side, and sham treatments, such as random and superficial needle insertion, have been utilized in trials. However, those methods might elicit a physiological response and are subsequently not ideal for use as a control. This descriptive study illustrates the construction of low-cost sham dry needles and evaluates their validity. Forty-two healthy asymptomatic subjects received either sham or real dry needling intervention to their right gluteal muscles and reported if they felt that the needle pierced the skin. They also graded the severity and qualified (sharp or dull) the pain associated with the intervention. The results showed that most of the subjects in both groups believed the needle penetrated the skin. The quantity of pain associated with the treatment was similar in both groups, but the quality assigned was different. The authors conclude that sham dry needling can be accomplished and used as a valid control treatment in dry needling research using these low-cost sham needles.

## 1. Introduction

Dry needling (DN) is described by the American Physical Therapy Association as “a skilled intervention that uses a thin filiform needle to penetrate the skin and stimulate underlying myofascial trigger points, muscular, and connective tissues for the management of neuromusculoskeletal pain and movement impairments” [1, 2].

As research into the efficacy of DN increases, the search for appropriate control groups and/or sham treatments intensifies as well. A valid control or sham treatment must be used in order to establish a cause and effect relationship of a treatment. A control group helps ensure that changes in the dependent variable (outcomes, such as “pain”) are due to changes in the independent variable (DN treatment or control/sham treatment) and not due to the therapeutic setting or chance. A true control group receives no intervention, while the experimental group receives some type of intervention. Sometimes a “waitlist group” is used as control group [3]. However, since DN is meant to be part of a comprehensive physical therapy treatment regimen and not to be administered as a stand-alone treatment, this option is not ideal. Another consideration in the search for an appropriate sham treatment is that it should offer some of the “treatment experience” that accompanies DN [4]. This is why some researchers choose to use the subject's contralateral side to the side that was dry needled as a control [5]. However, this solution is also less than optimal based on findings that needle electrodes can activate motor units on the contralateral side of the body [6]. There is also evidence that DN can affect pressure pain sensitivity of the contralateral side and at sites remote from the site that received treatment [7]. Other authors describe using needle insertion at random sites [8], in nontender points [9], or at the appropriate sites without reaching the necessary depth [10]. These methods may not be appropriate because evidence suggests that needle puncture, be it at a trigger point or not, could induce physiological responses including analgesia via descending inhibition [11] and thus skew the results. A placebo needle[12] has been described, where the needle disappears into the handle rather than penetrating the skin. These needles have the disadvantage of being relatively expensive in addition to not having a guide tube and are therefore of less interest to many physical therapists. However, a blunted needle that does not penetrate the skin is considered a reasonable sham treatment [12]. Tough et al. [4] described the making of a blunted needle, but very specialized equipment is needed. Tekin et al. [13] also used blunted needles as sham treatment but did not describe who manufactures or how one might construct such needles. With an invasive technique such as dry needling, a sham technique is probably not completely inert [14]. The process of palpation, preparing the site, discussion of treatment, and expected outcome, as well as the ritual of the needling itself, could all have some effect. In this study, the only part of the treatment that was changed was the actual insertion of a needle; thus all the other potential treatment effects remained the same. It is generally accepted that a treatment has nonspecific and specific effects [15]. The purpose of this report was to describe the construction and use of low-cost sham needles and evaluate their validity in DN research.

## 2. Materials and Methods

### 2.1. Methods of Validation

We used a sample of convenience including subjects from a university campus. Since this was a validation study of sham needles and not a trial on the effectiveness of DN, the subjects were not required to exhibit low back pain or a trigger point in the gluteal area. Forty-two volunteers (21 women and 21 men, average age 25.2 years, average BMI 23.9) participated in this study. This number is very similar to the number of subjects in a sham acupuncture needle validation study [4]. The only exclusion criteria were (1) fear of needles and (2) having been dry needled or acupunctured before. All the procedures were explained to the participants before written informed consent was obtained. The rights of the subjects were protected. All needling was performed by the PI, a licensed physical therapist who has been registered by the state of Utah to perform dry needling. The PI is an orthopaedic clinical specialist and a fellow of the American Academy of Orthopaedic Manual Physical Therapists (AAOMPT).

The subjects were told that two different types of needles would be used and that the needle may or may not penetrate the skin. The subjects were also told that they would be asked the following three questions [12] after the procedure: (1) Did the needle puncture skin? (2) How painful is the penetration of the needle through the skin on a scale from 0 to 10, where 0 represents no pain and 10 the worst pain imaginable (numeric pain rating scale, NPRS[16])? (3) Is the needle pain sharp or dull? These questions were very similar to another sham needle validation study [12]. Streitberger and Kleinhenz [12] concluded that the placebo needling was effective by comparing the answers to these questions between a group that received acupuncture and a group that received a type of placebo needling.

The subjects drew their group assignment in the form of a piece of paper from an opaque envelope. Their assignment specified either “Group A” (placebo/sham needle group) or “Group B” (DN group). To determine what the subjects felt during the actual needling the three questions mentioned above were asked to the subjects immediately following needling, after they rose from the treatment table. The subjects gave verbal responses.

This validation study was part of a greater study, where placebo needles were used (clinical trial unique protocol ID number 16225). The study was approved by the institutional review board of the PI's institution.

All subjects were positioned prone on a height-adjustable treatment table. The treatment site was always in the area of the right lower back, more specifically, about one to two inches distal to the posterolateral iliac crest, where the gluteal muscles are expected to lie. If a trigger point was palpable in the gluteal muscles, it was used for the site of needle insertion or sham treatment. The presence of a trigger point was determined by using flat palpation to determine if a site of localized tenderness could be found that also had tissue texture change and if a twitch response could be elicited with palpation.

**Figure 1:**
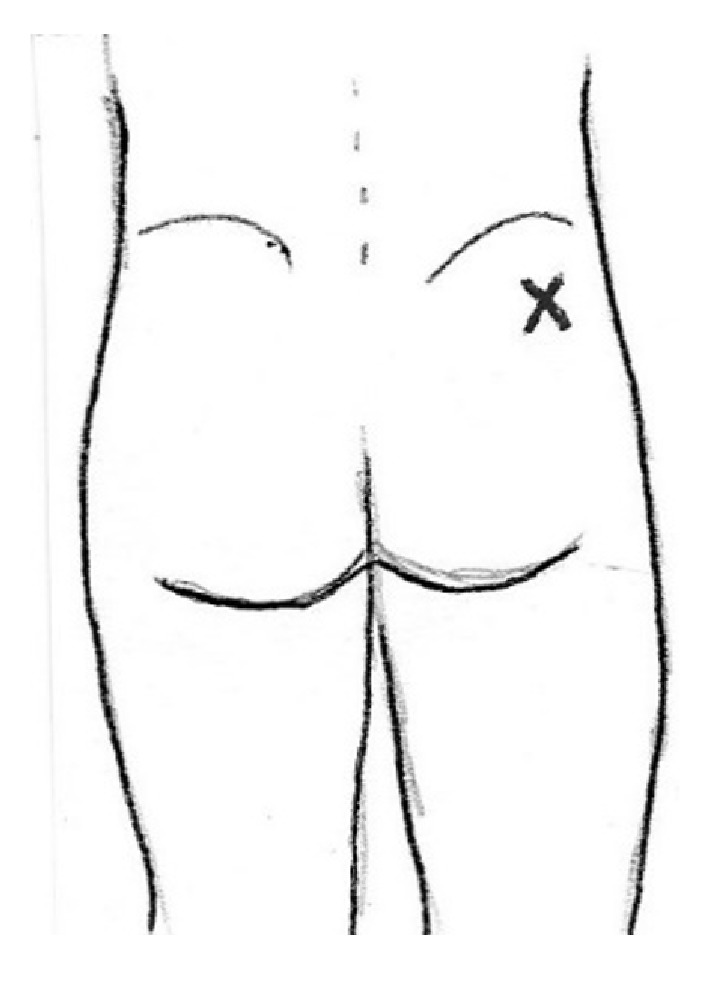
Site of needle intervention. A point in the posterior-lateral ilium was chosen for needle intervention. This area (indicated by x) can be the site of trigger points in the gluteus medius and minimus muscles.

If no trigger point was palpable, a point one to two inches distal to the posterolateral iliac crest, where gluteal trigger points are often located, was chosen (Figure 1).

Immediately prior to all interventions (sham or actual needle) the treatment site was cleaned with alcohol. The sham needles had been prepared prior to this validation study and were placed in a box that looked similar to the box with the actual needles. The actual dry needles were Seirin J-type needles (no. 8, 30 x 40mm) with guide tubes. Depending on the group assignment, the appropriate needle was taken out of its respective box and was opened within view of the subject. The guide tube was pressed against the tissue, which in turn was manually pressed against the posterolateral ilium, and the needle was allowed to drop against the skin. The handle was tapped briskly to break the skin and the tube was removed. The needle was slowly inserted into the underlying tissue until it either hit the bony backdrop (ilium) or reached a depth of about 3.5cm. The technique was an “in and out” one that was done twice in quick succession without the needle being fully withdrawn from the skin. No additional pistoning was performed. A twitch response was not required, since the intent of this study was to assess if subjects were able to distinguish between a sham and an actual dry needle, not if the treatment was effective. The needle was then discarded into a sharp container. The subject rose from the table, after which an investigator, who was blinded to the subject's group assignment, verbally asked the three questions outlined above. For the sham needles, the same approach was used with the exception of piercing the skin. The guide tube was pressed against the tissue and the sham needle was allowed to drop against the skin. The handle was tapped briskly but did not break the skin. The sham needle stayed within the guide tube and was pressed against the skin twice so as to mimic the quick “in and out” technique used for actual needling. Then the guide tube and sham needle were disposed of appropriately. Every intervention was performed by the PI to provide consistency of technique. The subjects were unable to view the actual needling procedure during treatment because of their positioning on the table.

### 2.2. Sham Needle Construction

**Step 1 .**

Seirin J-type needles (no. 8, 30 x 40mm) with guide tubes were utilized as the actual DN needles. In order to keep the looks of the sham needles as close as possible to the actual needles, we used the wrappings and guide tubes of slightly shorter Seirin J-type needles (no. 8, 30 x 30mm) for the making of the sham needles: the individual needle packages were carefully opened to the point where guide tubes (with needles) could be retrieved. Care was taken not to tear the paper wrapping. The needles were discarded in a sharps container.

**Step 2 .**

**Figure 2:**
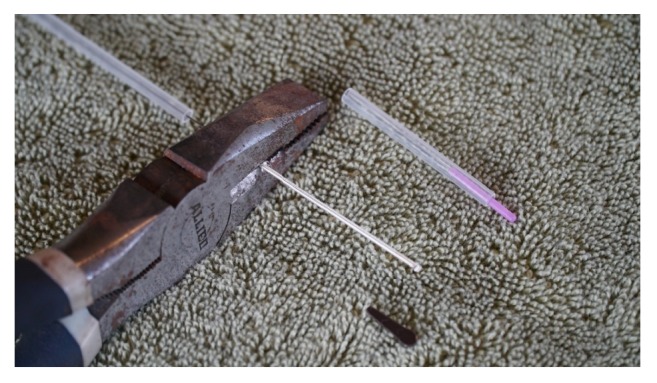
Cutting the 100mm needle at its root. The 100mm needles were removed partway out of the guide tube, taking care to leave the sharp end in the tube to avoid inadvertent poking. A pair of sharp wire cutters cut the needle at the junction of needle and shaft.

The handles of 50 x 100mm needles (Tai-Chi Brand, Lhasa OMS, Inc.) were used to construct the sham needles. They are about 50 mm long and fit in the guide tubes of 30mm needles (see Step 1). The 100mm needles were taken out of their wrappings and removed partway from their guide tubes. The guide tubes protected the distal, sharp ends of the needles to shield the investigator from being pricked. The long needles were cut with a wire cutter at the root, the point where the handle ends and the body of the needle begins (Figure 2).

**Figure 3:**
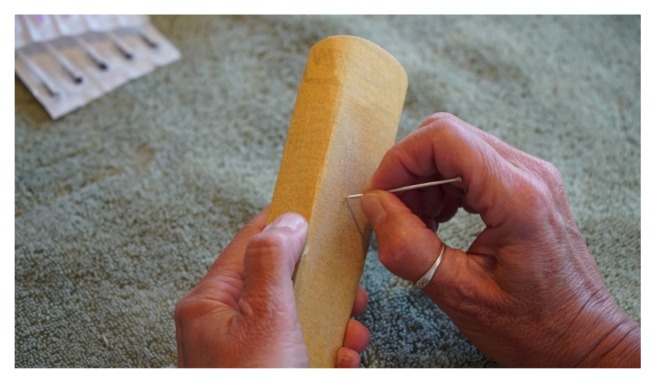
Sanding and smoothing the sham needle. The cut end of the handle was smoothed carefully with a sanding block so that no burrs or sharp edges could pierce the skin of the subjects.

The burred edges of the outside edges of the needles were manually smoothed and the tip leveled with 220-grit sand paper for about 4 seconds (Figure 3).

After that, they were tested for sensation compared to actual needles by pressing the tip gently against the PI's fingertip without breaking the skin. If sharp edges were felt, the tip was smoothed again with the same 220-grit sandpaper. Because the two types of needles used are of slightly different lengths and have a different type of handle, the subjects only saw the needle used for their treatment so that comparisons could not be made between needles by the subjects. The subjects were also unfamiliar with dry needling so they had no frame of reference for what the needle's appearance should be.

**Step 3 .**

**Figure 4:**
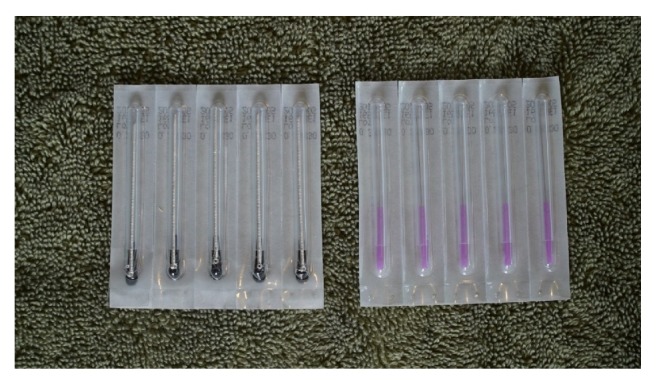
Sham and actual needles. The completed sham needles on the left. The length and packaging method look identical to “actual” dry needles.

The handle was inserted into the tubes of the 30mm needles and placed into the wrappings. The plastic tag was replaced to keep sham needles in place. The wrappings were smoothed out and glued at the edges (Figure 4).

### 2.3. Data Analysis

We conducted a two-sample test of proportions to test the first two null hypotheses, which were as follows: (1) the subjects cannot differentiate skin puncture between the actual or sham needles; (2) the quality of pain perceived (sharp/dull) is similar in both groups and a two-sample t-test was used to test the third hypothesis; (3) the rating of perceived pain associated with the sham or actual needles (on a scale from 0 – 10) is similar.

## 3. Results and Discussion

### 3.1. Results

Seventy-six percent of the subjects treated with actual needles correctly recognized skin puncture, and 86% of subjects in the sham group identified the sham treatment as “skin puncture”. Twenty-four percent of subjects in the actual needle group stated that no puncture occurred and 14% of subjects in the sham group stated that no puncture occurred (Table 1). To determine if differences exist between the two needles with regard to skin puncture we conducted a two-sample test of proportions which resulted in a p value of 0.69. Therefore, there is not enough evidence in the data to conclude that the proportion of individuals who thought they experienced skin puncture with the sham needle is different than that of the actual needle.

The pain sensation was perceived as mainly “sharp” in the sham treatment group (16 out of 21 subjects), while 11 out of 21 subjects characterized the needle sensation as “dull” in the dry needle group. Four subjects in the dry needle group, who reported that the needle did not penetrate the skin, chose to qualify the treatment sensation as neither sharp nor dull but instead chose “none”, although this was not an actual option (Table 1). These observations were not included when conducting a two-sample test of proportions which resulted in a test statistic of 4.9 and associated p value of 0.02. As a result, there is a statistically significant difference between proportion of subjects that felt sharp versus dull sensations between groups.

The average pain experienced with needling was slightly higher in the sham group compared to the treatment group, keeping in mind that it was still very low ≤ 1.5 on the NPRS for both groups (Table 1). Using a two-sample t-test to test for a difference in the average pain experienced resulted in a p value of 0.41, which leads to a similar conclusion to that regarding skin puncture.

Although the statistical procedures employed did not result in statistically significant differences, we cannot conclude that the proportions and means of interest are equal. To do this, tests of equivalence need to be considered. These tests reformulate the competing hypothesis so that rejecting the null hypothesis results in concluding that differences of proportions (or means) are within a prespecified range of values. For example, letting *μ*_A_ denote the average pain experienced when using the sham needle and *μ*_B_ denote the average pain for the real needle, the null and alternative hypotheses for a test of equivalence are as follows: H0: *μ*_A_ - *μ*_B_ < * Ɛ*_1_ or *μ*_A_ - *μ*_B_ > * Ɛ*_2_ versus H_1_  :* Ɛ*_1_  < *μ*_A_ - *μ*_B_ < * Ɛ*_2_, where* Ɛ*_1_ and* Ɛ*_2_ are specified upper and lower constants. The test of equivalence between proportions resulted in the difference of proportions associated with skin puncture being within 0.3 of each other (p value of 0.04) which implies that* Ɛ*_1_ = - 0.3 and* Ɛ*_2_ = 0.3.

For the test of equivalence associated with average pain between the two needles, we can conclude that the differences are between -0.5 and 1.0 (p value of 0.01). That is,* Ɛ*_1_ = - 0.5 and* Ɛ*_2_ = 1.0. It is known that the power associated with tests of equivalence is much lower than tests of difference counterparts. Because of this, a sample size larger than 21 will be needed in order to conclude that differences (either proportions or means) lie in a more precise interval.

## 4. Discussion

This report describes the procedure for performing sham dry needling as well as the construction of sham dry needles and their validation procedures, with the goal of facilitating sham-controlled dry needling research projects. An effective sham should appear to the subject as close to the actual treatment as possible. The packaging and wrapping of our sham device were very similar to the actual needle's packaging and wrapping, and the procedure immediately before and after use was the same. Our results indicate that subjects cannot differentiate between actual DN treatment and sham treatment, with regard to skin penetration and pain associated with needle insertion. The data support the hypothesis that the sham needles can provide a valid “fake treatment” or control condition, specifically during actual skin-piercing dry needling in the gluteal region. This method of sham needling provides the sensations of the same guide tube against the skin as is used for actual needling and the same quick “tap” that is used to insert the needle in the skin. These often serve to mask the sensation of actual skin-piercing. Thus, because the actual needle insertion into the skin is often painless, it should not be surprising that it was difficult for subjects to identify whether skin penetration occurred or not. Future studies could attempt to control even more for the sensation of needling by perhaps ensuring that the taps on the needle handles are exactly the same as well as perhaps seeing if maintaining versus removing the pressure of the guide tube against the skin affects the quality of the sham. The subjects in this study had never received dry needling before and the results could be different in subjects who had been treated with dry needling previously.

The needling technique in this study did not include “pistoning”, which is often performed during the procedure, especially if a local twitch response is not obtained on initial insertion. However, this particular technique is* not always* performed and because our subjects were novices to dry needling, they did not have an expectation of the needle pistoning. Future studies may want to test the influence of pistoning and “sham pistoning” as described by Mason et al. [17]. In that study, pistoning was done as part of the sham without puncturing the skin. It is yet to be determined how critical the local twitch response is to treatment effectiveness. Some studies have shown no difference in disability, pain intensity, or nociceptive sensitivity between groups who experienced a twitch response and those who did not [18].

The sham technique used in this study did not puncture the skin. While this is clearly different from actual needling, the fact that needle penetration did not occur means that this sham cannot produce the physiologic effects associated with tissue damage that occurs with needle penetration. We acknowledge that the sham technique described in this study does not have the physiologic effects of actual needling or cause a local twitch response. However, one needs to recognize that when a treatment such as dry needling is used, there are many other potential effects. This study only looked at the ability of a sham to cause a novice subject to believe that needle penetration did occur. The sham was successful in doing this; further studies should focus on other potential specific and nonspecific effects of dry needling to see if a sham for those effects can be effective. The physiologic effects of dry needling still need further study and until these effects are better understood, it will not be possible to determine if a sham elicits these physiologic effects or not. However, we believe that a sham that can cause a subject to believe that needle penetration has occurred has use in future dry needling investigations.

An interesting finding was the difference in which word the subjects chose to describe their respective intervention. The subjects were only able to choose between two words (sharp/dull). Most of the subjects in the sham treatment group chose the word “sharp” to describe the sensation they experienced during the intervention, while most of the subjects in the dry needle group chose the attribute “dull”. As already mentioned above, the pressure of the guide tube could have disguised the actual needle prick delivered in the dry needle group. Or it could be that the pressure of the tube without skin-piercing is more likely to be described as sharp. We modeled our validation study after Streitberger and Kleinhenz study[12], although that particular study used a specific acupuncture technique, while ours used a dry needling technique. The subjects in that study were only asked if they felt a “dull” sensation. This was done in order to capture “deqi”, which, according to traditional Chinese medicine, is a composite of unique sensations [19], often described as dull and achy. In dry needling the clinician is not attempting to elicit this deqi sensation, as in acupuncture [19]. We added the characteristic of “sharp” in our questioning to give the subjects another option of describing the sensation. The expectation of the patient in dry needling is that the needle feels “sharp” and penetrates. In our study, slightly more subjects in the sham group reported feeling skin puncture; thus we propose that our device could be an effective sham for dry needling. Further study regarding how patients describe sensations during dry needling may be helpful for clinicians and researchers.

It needs to be noted that none of the subjects in the sham needle group demonstrated any kind of skin bleeding, which would point to the breach of the skin.

We failed to reject our third null hypothesis, which stated that there is no difference in pain intensity between the two groups. It therefore appears that our sham was effective at eliciting a similar level of pain from the sham compared to the actual dry needling.

### 4.1. Limitations

Since we only used the needles in one anatomical area, we cannot generalize its validity to other parts of the body. We cannot also say that the same results would have been obtained if the sham needle was used in the line of vision. Additionally, because we tried to control for consistency of technique by only using one clinician in this study, the generalizability of the technique to other clinicians is limited. Our preliminary results support the notion that further research should be conducted to assess generalizability. Furthermore, this sham needle cannot be used for treatments where the needle is left* in situ*.

## 5. Conclusions

This report describes a procedure for conducting sham dry needling, the construction of simple and low-cost sham dry needles, and their validation. These sham needles and this procedure can be used as a valid control treatment when trying to establish the treatment effect of dry needling in the gluteal area.

## Figures and Tables

**Table 1:** Needle identification and perception of pain.

Treatment group	No skin penetration reported	Skin penetration reported	NPRS scores	Pain Sensation Dull /Sharp/none
Sham (n=21)	3	18	1.5 (0.9)	5/16/0
DN (n=21)	5	16	1.2 (1.3)	11/6/4

DN, dry needling; NPRS, numeric pain rating scale (0 to 10). Values are in mean (SD).

## Data Availability

This is a descriptive study; data associated with it are available from the primary author upon request.
